# MEN4, the MEN1 Mimicker: A Case Series of three Phenotypically Heterogenous Patients With Unique *CDKN1B* Mutations

**DOI:** 10.1210/clinem/dgac162

**Published:** 2022-03-22

**Authors:** Amanda Seabrook, Ayanthi Wijewardene, Sunita De Sousa, Tang Wong, Nisa Sheriff, Anthony J Gill, Rakesh Iyer, Michael Field, Catherine Luxford, Roderick Clifton-Bligh, Ann McCormack, Katherine Tucker

**Affiliations:** Cancer Genetics Laboratory, Kolling Institute, Royal North Shore Hospital, Sydney, NSW, 2065, Australia; The University of Sydney, Sydney, NSW, 2006, Australia; Cancer Genetics Laboratory, Kolling Institute, Royal North Shore Hospital, Sydney, NSW, 2065, Australia; The University of Sydney, Sydney, NSW, 2006, Australia; Endocrine and Metabolic Unit, Royal Adelaide Hospital, Adelaide, SA, 5000; South Australian Adult Genetics Unit, Royal Adelaide Hospital, Adelaide, SA, 5000, Australia; Adelaide Medical School, University of Adelaide, Adelaide, SA, 5000, Australia; The University of New South Wales, Sydney, NSW, 2052, Australia; The University of Western Sydney, Sydney, NSW, 2560, Australia; Department of Endocrinology, Prince of Wales Hospital, Sydney, NSW, 2064, Australia; Department of Endocrinology, Hornsby Ku-ring-gai Hospital, Sydney, NSW, 2077, Australia; The University of Sydney, Sydney, NSW, 2006, Australia; NSW Health Pathology, Department of Anatomical Pathology, Royal North Shore Hospital, Sydney, NSW, 2064, Australia; Cancer Diagnosis and Pathology Group, Kolling Institute, Royal North Shore Hospital, Sydney, NSW, 2064, Australia; Calvary Public Hospital, Canberra, ACT, 2617, Australia; Familial Cancer Service, Royal North Shore Hospital, Sydney, NSW, 2065, Australia; Cancer Genetics Laboratory, Kolling Institute, Royal North Shore Hospital, Sydney, NSW, 2065, Australia; The University of Sydney, Sydney, NSW, 2006, Australia; Cancer Genetics Laboratory, Kolling Institute, Royal North Shore Hospital, Sydney, NSW, 2065, Australia; The University of Sydney, Sydney, NSW, 2006, Australia; Department of Endocrinology, Royal North Shore Hospital, Sydney, NSW, 2064, Australia; Hormones and Cancer Group, Garvan Institute of Medical Research, Sydney, NSW, 2010, Australia; Department of Endocrinology, St. Vincent’s Hospital, Sydney, NSW, 2010, Australia; St. Vincent’s Clinical School, Faculty of Medicine, University of New South Wales, Sydney, NSW, 2010, Australia; Hereditary Cancer Service, Prince of Wales Hospital, Sydney, NSW, 2064, Australia; Prince of Wales Clinical School, University of New South Wales, Sydney, NSW, 2031, Australia

**Keywords:** multiple endocrine neoplasia type 4, MEN4, *CDKN1B* germline mutation, primary hyperparathyroidism, pituitary adenoma, pancreatic neuroendocrine tumor, atypical carcinoid tumor

## Abstract

**Context:**

Germline *CDKN1B* pathogenic variants result in multiple endocrine neoplasia type 4 (MEN4), an autosomal dominant hereditary tumor syndrome variably associated with primary hyperparathyroidism, pituitary adenoma, and duodenopancreatic neuroendocrine tumors.

**Objective:**

To report the phenotype of 3 unrelated cases each with a unique germline *CDKN1B* variant (of which 2 are novel) and compare these cases with those described in the current literature.

**Design/Methods:**

Three case studies, including clinical presentation, germline, and tumor genetic analysis and family history.

**Setting:**

Two tertiary University Hospitals in Sydney, New South Wales, and 1 tertiary University Hospital in Canberra, Australian Capital Territory, Australia.

**Outcome:**

Phenotype of the 3 cases and their kindred; molecular analysis and tumor p27^kip1^ immunohistochemistry.

**Results:**

Family A: The proband developed multiglandular primary hyperparathyroidism, a microprolactinoma and a multifocal nonfunctioning duodenopancreatic neuroendocrine tumor. Family B: The proband was diagnosed with primary hyperparathyroidism from a single parathyroid adenoma. Family C: The proband was diagnosed with a nonfunctioning pituitary microadenoma and ectopic Cushing’s syndrome from an atypical thymic carcinoid tumor. Germline sequencing in each patient identified a unique variant in *CDKN1B*, 2 of which are novel (c.179G > A, p.Trp60*; c.475G > A, p.Asp159Asn) and 1 previously reported (c.374_375delCT, p.Ser125*).

**Conclusions:**

Germline *CDKN1B* pathogenic variants cause the syndrome MEN4. The phenotype resulting from the 3 pathogenic variants described in this series highlights the heterogenous nature of this syndrome, ranging from isolated primary hyperparathyroidism to the full spectrum of endocrine manifestations. We report the first described cases of a prolactinoma and an atypical thymic carcinoid tumor in MEN4.

Multiple endocrine neoplasia includes several inherited syndromes associated with tumors in multiple endocrine organs, and sometimes nonendocrine tumors. Morbidity or mortality arises through excess hormone secretion, local growth and invasion, or metastasis. Multiple endocrine neoplasia type 1 (MEN1) syndrome resulting from germline heterozygous loss-of-function mutations in the tumor suppressor gene *MEN1* is well-recognized. The hallmark features of this syndrome include a high penetrance of primary hyperparathyroidism (PHPT), followed by pituitary adenoma, duodenopancreatic neuroendocrine tumors (DP-NET), and adrenal lesions.

Despite advances in genetic testing technology 5% to 25% of patients with clinically suspected MEN1 do not have a pathogenic variant within the *MEN1* coding region (1). Of these patients, technical limitations of molecular testing or failure to undertake detailed interrogation of the genome may account for the inability to identify the *MEN1* mutation. Sequencing of the promoter and untranslated regions and multiplex-ligand-dependent probe amplification to uncover gene deletions are required (2, 3). MEN1 phenocopies, which occur in 5-10% of cases, are an alternative explanation for not detecting an *MEN1* mutation on molecular analysis (4, 5).

Panel testing is now used in clinical practice to investigate familial hypercalcemia or syndromic/familial MEN1 syndrome at initial presentation, or in *MEN1*-mutation-negative patients. A typical multigene panel may include molecular analysis for germline mutations in hyperparathyroidism jaw tumor syndrome (*CDC73*); familial hypocalciuric hypercalcemia 1, 2, and 3 (FHH1, FHH2, and FHH3 from *CASR, GNA11, AP2S1*); familial isolated pituitary adenoma (with *AIP* accounting for 20%-30% of cases (6)); multiple endocrine neoplasia type 2 (*RET*); and, more recently, multiple endocrine neoplasia type 4 (MEN4, *CDKN1B)* (7, 8).

Thakker et al estimated that 3% of MEN1 mutation–negative patients harbor loss of function mutations in the cyclin-dependent kinase inhibitor 1B gene (*CDKN1B)* (1). Pathogenic variants within this gene result in the autosomal dominant condition MEN4 (OMIM #610755). MEN4 syndromically mimics MEN1 with some discernible differences. Primary hyperparathyroidism occurs most commonly with a seemingly older age of onset (9). The occurrence of anterior pituitary adenoma appears similar to MEN1, but DP-NETs are rare (9). Penetrance estimates are limited by the small number of cases described in the literature. Segregation studies in 1 kindred suggested penetrance may be near complete (9); however, other studies report unaffected variant carriers (10-12). The incidence of MEN4 syndrome is reported to be 1.5% to 3.7% in patients with a MEN1 related phenotype.

*CDKN1B* is a tumor suppressor gene comprising of 2 exons located on chromosome 12 (12p13.1) that encodes the nuclear protein p27^Kip1^ (14, 15). p27^Kip1^ is a cyclin-dependent kinase inhibitor limiting cell-cycle progression. Antimitogenic signals drive the formation of cyclin-dependent kinase inhibitor complexes with nuclear cyclin-dependent kinases D and E blocking their catalytic activity (16, 17). This prevents the transcriptional activation of genes involved in the transition from G1 to S phase (16, 18). Disruption of p27^Kip1^, resulting from loss of function mutations in CDKN1B, impedes its nuclear localization and binding with cyclin-dependent kinases (18-20). This is the key molecular mechanism driving MEN4 syndrome (19).

Genetic testing first identified homozygous *Cdkn1b* null alleles in a model rat that spontaneously developed multiple endocrine tumors as a recessive trait (anterior pituitary adenoma, phaeochromocytoma and paraganglioma, c-cell hyperplasia, and pancreatic islet cell hyperplasia) and was initially termed MENX because of the overlap of features with MEN1 and MEN2 (21). A heterozygous germline nonsense mutation in the *CDKN1B* gene was identified in a patient with a GH-secreting pituitary adenoma, parathyroid adenoma, and segregation within the family (11). A second patient described soon after was diagnosed with small cell neuroendocrine cervical carcinoma, an ACTH-secreting pituitary adenoma, and primary hyperparathyroidism (22). The 11th International Workshop on MENs subsequently named this MEN1-like syndrome MEN4 (23).

We aimed to describe and expand the phenotype of MEN 4 syndrome by presenting clinical, genetic, and immunohistochemical features of 3 unrelated patients who harbor pathogenic *CDKN1B* germline variants.

## Materials and Methods

### Ethics Approval

Informed consent was obtained from each individual and the research was approved by the Human Research Ethics Committee at Prince of Wales Hospital, Royal North Shore Hospital, and St Vincent’s Hospital, Sydney, and The Canberra Hospital, Canberra.

### Phenotype

Clinical information including presenting complaint, biochemical results, and radiological findings were obtained from the medical records.

### Germline Sequencing

Genomic DNA was extracted from blood using Qiagen DNAeasy extraction kits (Qiagen) for all patients.

#### Patient A

Using the Roche/NimbleGen SeqCap EZ Choice Library, Next Generation Sequencing on a custom panel incorporating 8 familial pituitary tumor syndrome genes (*AIP, CDKN1B, MEN1, PRKAR1A, SDHA, SDHB, SDHC*, and *SDHC)* was performed using Illumina’s HiSeq 2500 platform. Depth of coverage was > 30-fold in 97% of the targeted genomic region and > 100-fold in 91%. Sequencing data were processed according to Genome Analysis Toolkit’s (GATK) best practice. Sequencing reads were aligned to the human genome via Burrows-Wheeler Alignment and Novosort. Single-nucleotide variants and small insertions/deletions were identified and annotated with HaplotypeCaller v3.3 and Ensembl Variant Effector Predictor v74, respectively. Data were filtered and prioritized using an in-house platform.

#### Patient B

Next-generation sequencing (NGS) was performed incorporating 4 familial hypercalcemia syndrome genes (*MEN1*, *CDKN1B*, *RET*, and *CDC73*) using targeted capture (custom Roche SeqCap EZ capture “Roche 1k Disease v.6”) and sequenced on Illumina NextSeq Sequencing System. Sequences were aligned to the human reference genome (hg19) using Burrows-Wheeler Aligner (BWA mem). Variant calling was performed using GATK. Variant annotation was performed using Variant Studio v3, Alamut v2.11, and VariantGrid. Variants with a minor allele frequency > 1% or > 0.1% in population databases (ExAC, gnomAD) were excluded from the analysis.

#### Patient C

Targeted NGS of coding regions and splice sites of *MEN1* and *CDKN1B* was performed on the Illumina NextSeq500 with a targeted coverage of 700 reads/base. Seqliner v0.8 was used to generate aligned reads and call variants against the hg19 human reference genome. Copy number was analyzed using Gaffa v3.0 Targeted. PathOS v1.5 was used to annotate and transform variants to standard nomenclature and filter for rare, nonsynonymous variants within 20 bp of coding regions.

Sanger sequencing was used to confirm the identified variants within CDKN1B. Variant nomenclature is according to Human Genome Variant Society nomenclature v20.05 (24) using the reference sequence CDKN1B (NCBI:NM_0004064.4;NG_016341.1) (25). Interpretation of variant pathogenicity is according to American College of Medical Genetics (ACMG) guidelines (26).

### Tumor Sequencing

Tumoral DNA was extracted from formalin-fixed paraffin embedded tumor samples using Qiagen DNAeasy blood and tumor extraction kit (Qiagen, Germany). Tumor specimens available for analysis were as follows: parathyroid adenoma, adrenal cortical adenoma, and pancreatic neuroendocrine tumor from patient A; parathyroid adenoma from patient B; and atypical thymic carcinoid tumor from patient C. DNA libraries were prepared and sequenced on a MiSeq platform using a custom hereditary endocrine tumor gene panel (TruSeq Custom Amplicon Assay, Illumina). FASTQ files were generated for each sample, and alignment of reads (banded Smith-Waterman algorithm) and variant calling (GATK) was processed by MiSeq Reporter v2.0, Illumina. Annotation of functional consequences to variant calls was performed using ANNOVAR (version2013Jul). Output was filtered to analyze relevant coding regions (CDKN1B to assess for loss of heterozygosity [LOH], MEN1, and ATRX), to remove untranslated regions and synonymous single nucleotide polymorphisms, include read depth ≥ 30 and include variants with a population variant allele frequency < 0.001 or not reported in the population database gnomAD (27). Visualization of reads was performed using IGV v2.1. The Catalogue of Somatic Mutations in Cancer was used to categorize somatic variants with regard to pathogenicity (28).

### p27^Kip1^ Immunohistochemistry

Immunohistochemistry for p27^Kip1^ was performed on archived formalin-fixed paraffin embedded tissue using a commercially available mouse monoclonal antibody (RRID: AB_11190322, https://scicrunch.org/resources/Any/search?q=AB_11190322&l=AB_11190322, clone SX53G8, predilute, catalog number LS-C389523, Ventana Medical Systems, Tucson, AZ, USA) on an automated staining platform—the Ventana BenchMark Ultra (Ventana Medical Systems) using the manufacturer’s protocol.

## Results

Clinical information and the outcome of molecular testing are summarized for each patient in Table 1. Detailed laboratory results are provided in Table 2.

**Table 1.** Clinical features and outcome of molecular testing

	Tumor (age, y)	Family history	Germline pathogenic variant	LOH	Somatic pathogenic variant
Patient A	Multigland PHPT; single adenoma (33 y), second adenoma (45 y) Prolactinoma (39 y) Multifocal pancreatic NET (47 y) Benign cortical adrenal adenoma (47 y)	First-degree relative with PHPT (genetic testing not undertaken)	*CDKN1B* (c.179G > A, p.Trp60Ter)	No	No
Patient B	PHPT (26 y)	No	*CDKN1B* (c.475G > A, p.Asp159Asn)	No	*ATRX* (c.6406G > A, p.Asp2136Asn)
Patient C	Atypical thymic carcinoid tumor (31y)	Second-degree relative who underwent “neck surgery”	*CDKN1B* (c.374_375delCT, pSer125*)	No	*MEN1* (c.974C > T, p.Pro325Leu)

Abbreviations: LOH, loss of heterozygosity; NET, neuroendocrine tumor; PHPT, primary hyperparathyroidism.

**Table 2.** Results of laboratory investigations

	Patient A	Patient B	Patient C
PTH	1.5 × ULN (12.4 pmol/L, rr 0.8–8.0)	3.0 × ULN (20 pmol/L, rr 1.5-6.9)	1.2 × ULN ( 8.8 pmol/L, rr 1.6-7.2)
Corrected calcium	1.9 × ULN (2.92 mmol/L, rr 2.16-2.45)	1.2 × ULN (3.06 mmol/L, rr < 2.55)	Normal
25OHD	Normal	Normal	0.4 × LLN (24 nmol/L, rr > 50)
TSH	Normal	Normal	Normal
Prolactin	3.8 × ULN (1898 mIU > L, rr 50-500)	Normal	Normal
IGF-1	1.0 × ULN (251 µg/L, rr 93-245)	Normal	1.2 × ULN (49 nmol/L, rr 8-42)
GH	Normal	Normal	Normal
ACTH	Normal	Normal	1.5 × ULN (21 pmol/L, rr < 14)
Cortisol	1.3 × ULN	Normal	Normal
Morning (600 am)	(699 nmol/L, rr 100-535) (initial) Normal (490 nmol/L) (repeat)	-	-
Midnight salivary	1.1 × ULN (3.6 nmol/L, rr < 3.2)	-	-
24-h urinary excretion (preoperative)	2.3 × ULN (624 nmol/L, rr < 270)	-	2.9 × ULN (438 nmol/24h, rr < 150)
24-h urinary excretion (postoperative)	-	-	Normal
1 mg DST	1.2 × ULN (62 nmol/L, rr < 50)	-	5 × ULN (249 nmol/L, rr < 50)
DHEAS	1.6 × ULN (11.3 µmol/L, rr 1.0-7.0)	-	-
Androstenedione	Normal	-	-
HbA1c%	Normal	-	1.2 × ULN (6.7%, rr < 5.6%)
CgA	Normal	Normal	Normal
Gastrin	Normal	Normal	Normal
5HIAA			
Preoperative	-	-	1.5 × ULN (53 µmol/24 h, rr < 35)
Postoperative	-	-	Normal
c-peptide	-	-	1.5 × ULN (2.3 nmol/L, rr 0.4-1.5)
Insulin	-	Normal	N/A
VIP	Normal	Normal	N/A
Glucagon	Normal	Normal	N/A
Pancreatic polypeptide	Normal	1.4 × ULN (78.1 pmol/L, rr < 55)	N/A

Abbreviations: -, not performed; 5HIAA, 5-hydroxyindoleacetic acid; CgA, chromogranin A; DHEAS, dehydroepiandrosterone sulfate; DST, dexamethasone suppression test; HbA1c, glycated hemoglobin; N/A, performed but not available; rr, reference range; ULN, upper limit of normal; VIP, vasoactive intestinal peptide.

### Patient A

#### Clinical details

The proband (II.2, Fig. 1.1) presented aged 32 years with PHPT (corrected calcium 2.92 mmol/L, normal range 2.16-2.45 mmol/L; PTH 12.4 pmol/L, normal range 0.8-8.0 pmol/L) and a left superior parathyroid adenoma was resected via minimally invasive surgery. More than a decade later, aged 45 years, a recurrence of PHPT was diagnosed (corrected calcium 2.69 mmol/L, normal range 2.1-2.6 mmol/L; PTH 104 pg/mL, normal range 15-68 pg/mL) and a second parathyroid adenoma was resected. Hypercalcemia resolved postoperatively on both occasions. End-organ complications of PHPT included osteopenia (L1-L4 T-score -0.3 SD and left femoral neck T-score -1.5 SD on dual-energy X-ray absorptiometry) and asymptomatic nonobstructive renal calculi diagnosed radiologically.

**Figure 1.**
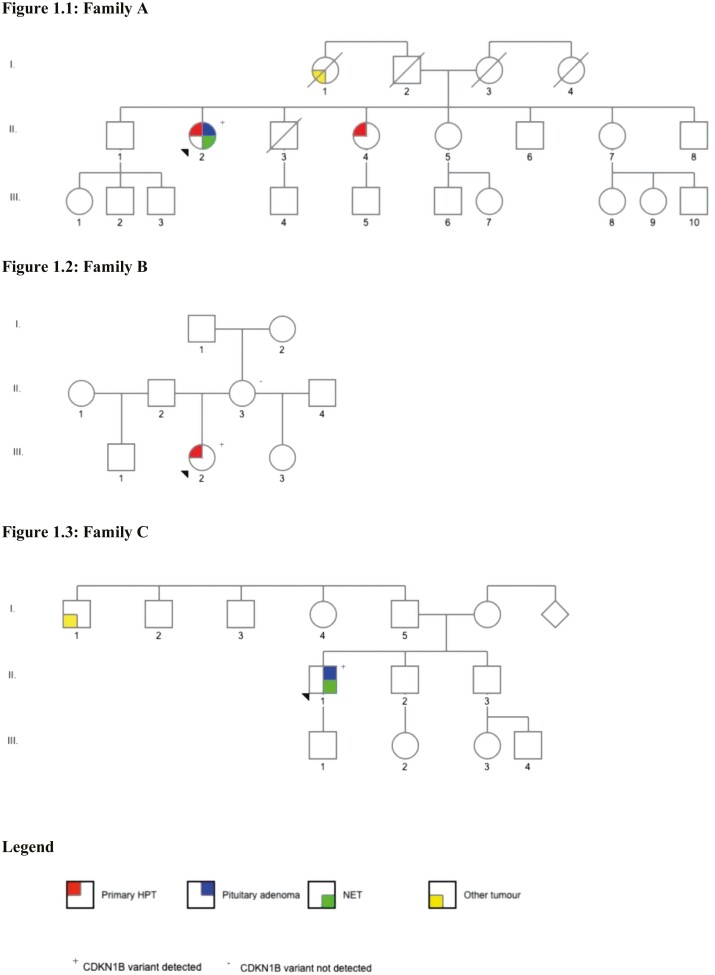
Pedigrees. (1.1) Family A. (1.2) Family B. (1.3) Family C.

At age 39 years following the development of recurrent tonic-clonic seizures a cerebral ^18^F-fluorodeoxyglucose-positron emission tomography (PET) scan was performed to evaluate for an epileptogenic focus. This revealed increased tracer uptake in the region of the pituitary gland. Further investigation with contrast-enhanced pituitary magnetic resonance imaging (MRI) identified an 8 × 6 × 3-mm hypointense nodule in the right aspect of the adenohypophysis consistent with a pituitary microadenoma. A pituitary hormone panel found isolated hyperprolactinemia (prolactin 1898 mIU/L, normal range 50-500 mIU/L), from which she was symptomatic with oligomenorrhea. She was started on cabergoline 0.5mg weekly with normalization of prolactin within 4 months.

At age 43 years, the patient was investigated for hypercortisolemia in the context of a 16kg weight gain and plethoric facies. ACTH was normal (1.9pmol/L, normal range < 10.8pmol/L) and cortisol was elevated (699pmol/L, normal range < 539pmol/L). Midnight salivary and 24-hour urinary free cortisol (UFC) levels were both elevated (3.6nmol/L, normal range < 3.2nmol/L; and 624nmol/L, normal range < 270nmol/L, respectively). An overnight 1-mg dexamethasone suppression test failed to suppress cortisol below 50nmol/L. On repeating the 24-hour UFC, salivary cortisol and dexamethasone suppression test results were normal, raising the possibility of cyclical Cushing’s syndrome.

**Figure 2.**
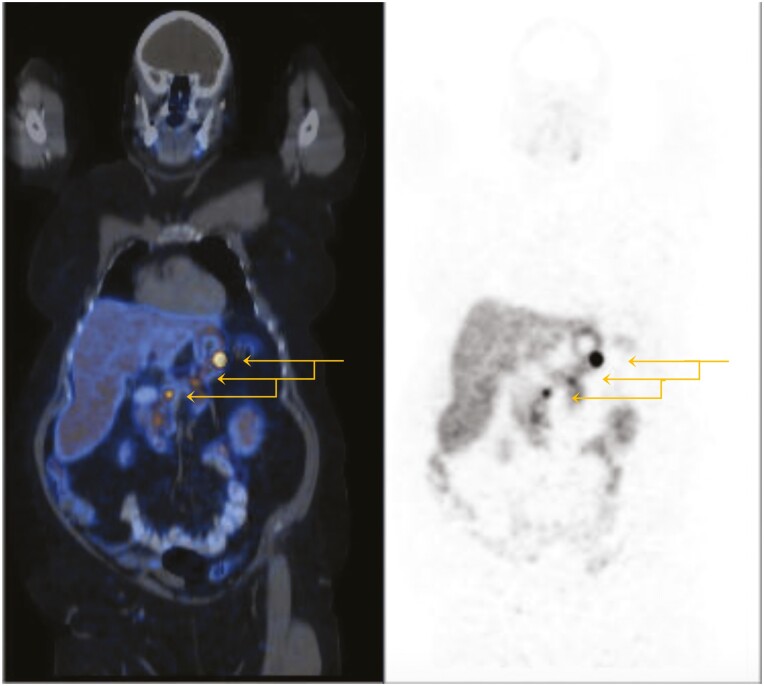
Functional imaging. ^68^Ga-DOTATATE-PET of patient A.

In the context of young-onset multiglandular primary hyperparathyroidism and a microprolactinoma, genetic testing was undertaken and no pathogenic variant was identified in *MEN1.* Based a clinical diagnosis of MEN1 syndrome, a screening MRI of the abdomen was performed; 2 adrenal lesions were found (on the left measuring 59 × 49 mm [-5HU] and on the right 26 × 21 mm [-17HU]) and multiple pancreatic lesions in the body and tail of the pancreas, with the largest measuring 15 × 13 × 10 mm. The pancreatic lesions were avid on ^68^Ga-DOTATATE-PET (Fig. 2). An adrenal hormone panel (plasma metanephrines, renin, aldosterone, cortisol) and a limited pancreatic hormone panel (gastrin, pancreatic polypeptide, vasoactive intestinal polypeptide) were normal.

Left adrenalectomy was undertaken to remove the large left nonfunctioning adrenal nodule based on the size. There was no evidence of hypercortisolism perioperatively. Distal pancreatectomy was performed to resect the multiple pancreatic lesions. Histopathology of the left adrenal nodule showed a 53mm encapsulated tumor consistent with an adrenal cortical adenoma. Histopathology of the pancreas showed 3 well-differentiated neuroendocrine tumors measuring 2, 4, and 11mm each with Ki67 < 1%. A repeat ^68^Ga-DOTATATE-PET scan 12 months following surgery showed a possible recurrence at the pancreatic resection margin and new lesions in the neck and body of the pancreas. The patient underwent total pancreaticoduodenectomy; histopathology identified 2 neuroendocrine tumors measuring 13 × 12 × 12 mm in the body of pancreas and 5 × 5 × 5 mm in the neck. Ki67 of each tumor was < 1%.

Additional diagnoses include polycystic ovarian syndrome and antiphospholipid syndrome. Her family history is notable for a sister (II.4, Fig. 1.1) with a parathyroid adenoma diagnosed at age 50 years. Family members have declined predictive testing.

#### Germline and tumor sequencing

The patient was recruited into the Familial Pituitary Tumour Syndrome study, a cross-sectional study aimed at identifying germline variants in patients diagnosed with pituitary tumors ≤ 40 years of age and/or other personal/family history of endocrine neoplasia (29). Germline sequencing identified a *CDKN1B* (c.179G > A, p.Trp60*) nonsense variant resulting in a premature stop codon in exon 1, which is predicted to lead to nonsense-mediated decay of CDKN1B mRNA with loss of protein function (Fig. 3.1). This variant has not been reported in the scientific literature to date or in population (gnomAD v2.1.1, 1000Genomes (30)) or variant (ClinVar (31), LOVD (32), HGMD (33)) databases. In silico tools predict this variant to be pathogenic (CADD score 38). As per ACMG guidelines the variant is classified as “likely pathogenic” (PVS1, PM2).

**Figure 3.**
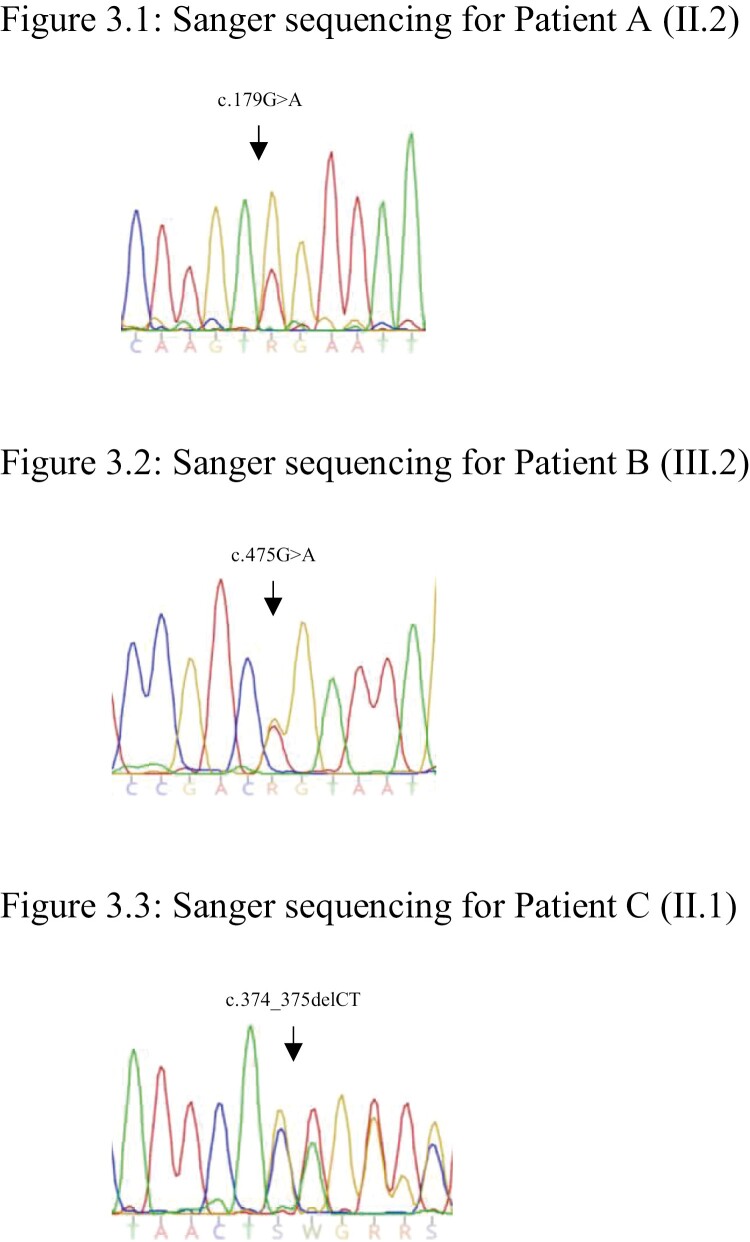
Germline CDKN1B electropherograms. (3.1) Sanger sequencing for patient A. (3.2) Sanger sequencing for patient B. (3.3) Sanger sequencing for patient C.

NGS of parathyroid adenoma, adrenal cortical adenoma, and pancreatic neuroendocrine tumor identified the same *CDKN1B* (c.179G > A, p.Trp60*) variant as was present in the germline. There was no LOH. No additional “pathogenic” or “likely pathogenic” variants were identified.

#### p27^Kip1^ immunohistochemistry

Immunohistochemistry of the resected parathyroid adenoma showed loss of staining for p27^Kip1^ consistent with an acquired second hit in p27^Kip1^ resulting in subsequent loss of expression in the neoplastic cells (Fig. 4.1). Loss of p27^Kip1^was also seen in the adrenal cortical adenoma but without a positive internal control limiting its significance. The pancreatic NET demonstrated loss of expression with positive expression in the internal controls in the stroma (Fig. 4.2). However, because a similar pattern of loss of expression was identified in all non-neoplastic islets from the same patient, this pattern of staining is considered noncontributory (ie, p27^Kip1^ loss may be a feature of islet cell differentiation).

**Figure 4.**
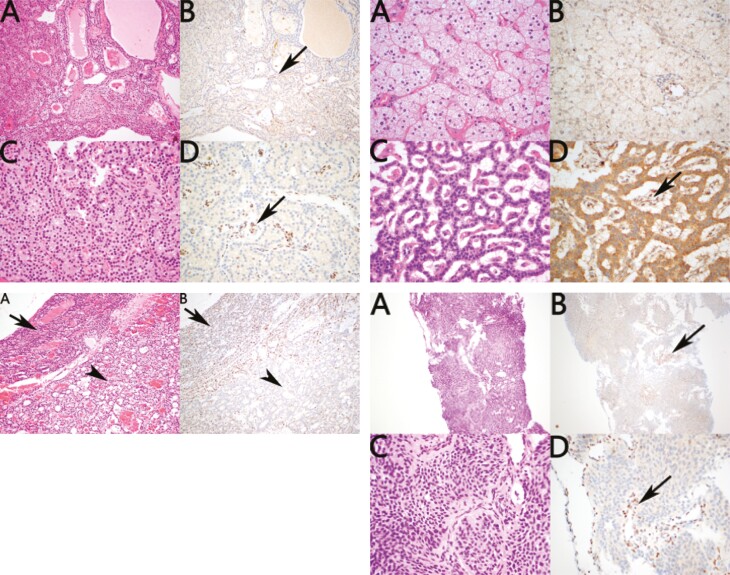
Immunohistochemistry. (4.1, top left) p27^kip1^ immunohistochemistry of parathyroid adenoma from patient A. (4.2, top right) p27^kip1^ immunohistochemistry of adrenal cortical adenoma and pancreatic neuroendocrine tumor from patient A. (4.3, bottom left) p27^kip1^ immunohistochemistry of parathyroid adenoma from patient B. (4.4, bottom right) p27^kip1^ immunohistochemistry of the thymic neuroendocrine tumor from patient C.

### Patient B

#### Clinical details

The proband (III.2, Fig. 1.2) was diagnosed with primary hyperparathyroidism aged 26 years (peak corrected calcium 3.06mmol/L, normal range 2.5-2.55mmol/L; PTH 20pmol/L, normal range 1.5-6.9pmol/L). Parathyroid sestamibi imaging demonstrated a hyperfunctioning right inferior gland, and an equivocal focus on the left concerning for multigland disease. The highly avid gland was resected with resolution of hypercalcemia; there is no evidence of recurrence 3 years later.

The diagnosis of primary hyperparathyroidism under the age of 40 years prompted genetic testing for hereditary causes of hypercalcemia, including MEN1, MEN4, and hyperparathyroidism jaw tumor syndrome. A mutation search in MEN1 and CDC73 was negative; a pathogenic variant was identified in CDKN1B. The diagnosis of MEN4 syndrome resulted in further investigation for additional syndromic features. MRI of the pituitary did not identify a pituitary adenoma. Pituitary and pancreatic hormone measurements were normal. Imaging of the pancreas for a nonfunctioning NET has not yet been performed.

The patient has a medical history of polycystic ovary syndrome and depression. There is no known family history of endocrine or related neoplasia; however, the details of her paternal family history are unknown. Her mother (II.3, Fig. 1.2) underwent predictive testing for the *CDKN1B* pathogenic variant and was wild-type.

#### Germline and tumor sequencing

Germline sequencing identified a novel heterozygous *CDKN1B* (c.475G > A, p.Asp159Asn) missense variant involving the final codon of exon 1 (Fig. 3.2). This variant has not been reported in scientific literature to date and is not listed in population (gnomAD v2.1.1, 1000Genomes (30)) or variant (ClinVar (31), LOVD (32), HGMD (33)) databases. In silico tools predict the variant to remove a splice donor site (Alamut version 2.7) and to be “probably damaging” (PolyPhen) and “deleterious” (SIFT score 0.005, CADD score 29.1). Per ACMG criteria, the variant is classified “likely pathogenic” (PVS1, PM2).

NGS of parathyroid adenoma identified the identical *CDKN1B* (c.475G > A, pAsp159Asn) variant as was present in the germline with no LOH. A somatic *ATRX* (c.6406G>A, p.Asp2136Asn) missense variant was identified and has been reported in glioma (astrocytoma grade IV) and endometrioid carcinoma (34, 35). In silico analysis predicted the variant to be deleterious (SIFT score 0.002, PolyPhen 1.0, MutationTaster 1.0).

#### p27^Kip1^ immunohistochemistry

Immunohistochemistry of the single parathyroid adenoma for p27^Kip1^ demonstrated somewhat decreased expression in the neoplastic cells compared with non-neoplastic cells without complete absence of staining (Fig. 4.3). That is, although this pattern of staining supported the likelihood of decreased expression of p27^Kip1^, it was not definitive.

### Patient C

#### Clinical details

The proband (II.1, Fig. 1.3) presented aged 31 years with an atypical thymic carcinoid tumor causing ectopic Cushing’s syndrome.

This diagnosis was made in the context of a hospital admission for severe hypertension (SBP 199/80 mm Hg) and clinical features consistent with cortisol excess (rounded facies, abdominal striae, supraclavicular/interscapular fat pads). The patient was started on 2 antihypertensive agents. Type 2 diabetes mellitus was also diagnosed and managed with metformin. Investigations into secondary causes of diabetes were performed and identified ACTH-dependent hypercortisolism with an elevated 24-hour UFC (438nmol/24 hr, normal range < 150 nmol/24 hr), lack of cortisol suppression on a 1-mg overnight dexamethasone suppression test, and an elevated ACTH (21 pmol/L, reference range < 14pmol/L). Contrast-enhanced MRI of the pituitary reported a 7mm focus of “differential low enhancement” on the right side of the pituitary suggestive of a pituitary microadenoma. However, bilateral inferior petrosal sinus sampling confirmed an ectopic source of ACTH, with central-to-peripheral plasma ACTH gradient of < 2.0 before corticotropin-releasing hormone administration and < 3.0 after corticotropin-releasing hormone.

Computed tomography imaging identified an ovoid superior mediastinal mass with a slightly irregular border measuring 66 × 45 mm that abutted the brachiocephalic vein and aortic arch. Histopathology from a CT-guided core biopsy of the superior mediastinal mass was consistent with a thymic neuroendocrine tumor. The tumor was resected via thoracotomy, which on histopathology was a 59mm atypical carcinoid tumor (pT3pN0M0). After recovering from a postoperative pleural effusion, exogenous glucocorticoid replacement was weaned, and UFC normalized. His diabetes resolved. He remains on single-agent antihypertensive treatment. Postoperative radiotherapy was administered to the mediastinum (3-dimensional conformal radiation therapy, 60 Gy/30 fractions). Now 2 years following his diagnosis, he remains without evidence of clinical, biochemical, or radiological recurrence.

The patient has no additional medical history. He reported a paternal aunt (I.1 Fig. 1.3) with a neck tumor resected between the ages of 30 and 40 years. His mother and brother have a diagnosis of hypertension. No family members have undergone predictive testing as his immediate and extended family live overseas.

#### Germline and tumor sequencing

Germline sequencing identified a heterozygous variant in exon 1 of *CDKN1B* (c.374_375delCT, p.Ser125*), with the deletion of 2 bases resulting in a premature stop codon predicted to result in nonsense-mediated decay (Fig. 3.3). This variant has not been reported in population (gnomAD v2.1.1, 1000Genomes (30)) or variant (ClinVar (31), LOVD (32), HGMD (33)) databases. The variant has been detected in an individual with multiglandular PHPT and DP-NET with complete loss of nuclear p27^kip1^ expression in the parathyroid adenoma on immunohistochemistry (12). Per ACMG guidelines, the variant is classified as “pathogenic” (PVS1, PS3, PM2)

NGS of the atypical thymic carcinoid tumor identified the identical *CDKN1B* (c.374_375delCT) variant as was detected in the germline with no LOH. A somatic *MEN1* (c.974C > T, p.Pro325Leu) missense variant was detected. This variant has previously been reported in sporadic parathyroid adenoma (36). In silico analysis predicted the variant to be “deleterious” (SIFT score 0.002, PolyPhen 1.0, MutationTaster 1.0).

#### p27^Kip1^ immunohistochemistry

Immunohistochemistry of the thymic atypical carcinoid for p27^Kip1^ was completely negative in all neoplastic cells, with preserved diffuse strong expression in the non-neoplastic cells, which served as an internal positive control (Fig. 4.4). This pattern of staining is in keeping with an acquired second hit in p27^Kip1^ with subsequent loss of expression in the neoplastic cells.

## Discussion

We describe three unrelated cases of MEN4 syndrome resulting from different germline *CDKN1B* null variants, including two novel variants. All patients developed an endocrine tumor at a young age (with one patient developing multiple endocrine tumors), prompting genetic testing for a hereditary endocrine tumor syndrome. We report the first cases of prolactinoma and a thymic carcinoid tumor in MEN4 syndrome. These three cases and their kindreds emphasize the phenotypic heterogeneity of MEN4 syndrome.

Our three cases add to more than 34 previously reported cases of MEN4 syndrome, with 21 unique mutations (9, 37-39). Of the various endocrine tumors in MEN4, parathyroid adenomas are most common (either single or multiple), followed by other endocrine tumors (pituitary and adrenal adenoma, DP-NET), and rarely nonendocrine tumors. Primary hyperparathyroidism develops in most, if not all, carriers of a *CDKN1B* mutation with an estimated penetrance approaching 90% to 100% (9, 19). Frederikson et al presented an extensive family segregation study with 13 individuals harboring a *CDKN1B* pathogenic variant; all developed primary hyperparathyroidism (9). Although this resembles MEN1 syndrome, biochemical aberrations appear milder, are often asymptomatic, and with an onset later in life (mean age of diagnosis 56 years) (9, 13). The youngest age of onset reported is 15 years (10), and patient A adds evidence that multiglandular PHPT does occur (12).

14 cases of pituitary tumors have been described in the literature, four nonfunctioning (9, 40), five with acromegaly (11, 39, 41, 42), five with Cushing’s disease (9, 22, 38), and the first described case of a prolactinoma resulting from a pathogenic *CDKN1B* mutation has now been identified in this series. The penetrance of pituitary adenoma is estimated to approach 30% (38). Of note, Chasseloup et al screened a predominantly pediatric cohort with Cushing’s disease and identified 3 disease-causing (pathogenic/likely pathogenic) variants in early adolescence with the youngest age of disease onset at 9 years (38).

Nine cases of DP-NET have been described in MEN4 syndrome, with the youngest age of onset at 42 years (43), of which three cases were gastrinomas and six nonfunctioning (12, 19, 43, 44). Although DP-NET may result in a high degree of morbidity and mortality in MEN1 patients, the natural progression in MEN4 has not been established.

These key features of MEN4 syndrome contrast with MEN1 syndrome whereby the penetrance of primary hyperparathyroidism approaches 100% by age 50 years with a high rate of recurrent disease (>50% by 12 years) (45, 46). The penetrance of pituitary adenoma in MEN1 is ~40% with lactotroph adenoma occurring most frequently (20%), and somatotroph, gonadotroph, and clinically nonfunctioning adenoma occurring infrequently (~5% each). Corticotroph (2%) and TSHomas rarely develop (45). DP-NET penetrance may be as high as 90% with gastrinoma occurring in 30% to 40% of patients, followed by nonfunctioning adenoma in 20% to 55% and insulinoma in 10% (45, 47).

Less frequent endocrine lesions occurring in MEN1 syndrome include adrenocortical tumors, gastric NET, bronchopulmonary NET, thymic NET, and pheochromocytoma with an estimated prevalence of 40%, 10%, 2%, 2%, and < 1%, respectively (45). Bilateral adrenal nodular hyperplasia, primary bilateral adrenocortical hyperplasia, and adrenocortical carcinoma have been described in MEN1 syndrome. In comparison, adrenal nodules (including patient A from this study) have been described in patients with MEN4 syndrome (13), and bronchial (40) and neuroendocrine cervical carcinoma have occurred in individual cases (22). Thymic NETs are already known to have a strong association with hereditary endocrine syndromes, with 25% occurring in the context of MEN1 syndrome (48, 49). MEN1-associated thymic NETs are highly lethal. Lifelong radiological screening because of the primarily nonfunctional nature of these tumors is recommended and risk-reducing transcervical thymectomy at the time of parathyroidectomy is suggested (8). Patient C is the first described case of a thymic neuroendocrine tumor in a patient in MEN4 syndrome. Whether a high mortality rate will transpire within this syndrome is yet to be seen.

The small number of cases and a paucity of segregation studies limits confidence in recommending surveillance guidelines for primary tumors. Considering our findings in the context of previously described MEN4 cases in the literature, screening for primary hyperparathyroidism biennially from age 15 years, pituitary adenoma from 10 years and DP-NET from age 30 years (10 years earlier than the youngest described case because of the high degree of morbidity associated with NETs) could be considered. MRI (to coincide with pancreatic imaging) to detect thymic NET should also be incorporated.

In our three cases, tumor testing failed to demonstrate a second somatic event in the wild-type allele, which is in keeping with the literature. Haploinsufficiency for *CDKN1B* appears sufficient for tumors to develop (50). Murine studies in 1998 by Fero et al first identified this, showing both p27^Kip1^ nullizygous and p27^Kip1^ heterozygous mice challenged with gamma-irradiation or a chemical carcinogen were predisposed to developing tumors in multiple tissues, including pituitary tumors (51). Following on from these findings, Teixeira et al highlighted the role of p27^Kip1^ in hampering aberrant somatotroph proliferation in p27^Kip1^ deficiency with even partial reductions in p27^Kip1^ augmenting pituitary oncogenesis (52). Within MEN4 syndrome, LOH has been found in a minority of tumor samples (9, 12, 22) with inconsistency between tumor types and between tumors from a single patient (44). As such, CDKN1B shares similarities with *p53*, *NF1*, and *PTEN* as “early generation” haplo-insufficient tumor suppressor genes (50).

Silencing of the wild-type allele by somatic mutations in alternative genes is a proposed trigger for tumor development in haploinsufficiency (19). Examples of such genes specific to endocrine tumors include other CDK family members (*CDKN2B, CDKN2C, CDKN1A*) and, as identified in the thymic carcinoid tumor from patient C, *MEN1*. *ATRX* was identified in the parathyroid adenoma tissue from patient B. Mutations in *ATRX* are associated with alternative lengthening of telomeres and have been shown to be associated with clinically aggressive behavior in *SDHB* mutated phaeochromocytoma and paraganglioma (53). *ATRX* and *MEN1* genes are both involved in chromatin remodelling and have been identified as the most frequent somatic mutation in pancreatic NETs (54, 55). Expanding tumor sequencing in MEN4 syndrome may identify genotype/phenotype correlations with these other well-recognized tumor suppressor genes.

A key limitation of the study is the absence of segregation studies with only the single unaffected first-degree relative of patient B undergoing predictive testing. All three patients are in the third and fourth decades of life and potentially are yet to develop further syndromic manifestations.

## Conclusion

MEN4 syndrome demonstrates the hallmark features of many hereditary endocrine cancer syndromes: tumor multifocality and phenotypic heterogeneity. Index cases with features consistent with MEN1 should be considered for molecular testing for a *CDKN1B* mutation either as part of panel testing for MEN1 phenocopies or if genetic testing has not identified a mutation in *MEN1*. Simultaneous testing of *MEN1* and *CDKN1B* in patients with an MEN1 phenotype is increasingly applicable given improved access to NGS. As with many other rare hereditary tumor syndromes, the heterogenous pattern of disease presentation has hampered the development of syndrome-specific management guidelines. As such, the frequency of tumor specific biochemical and radiological surveillance in MEN4 syndrome is yet to be established.

## Data Availability

All data analyzed during this study are included in this published article.

## Abbreviations

ACMG: American College of Medical Genetics
DP-NET: duodenopancreatic neuroendocrine tumor
GATK: Genome Analysis Toolkit
LOH: loss of heterozygosity
MEN1: multiple endocrine neoplasia type 1
MEN4: multiple endocrine neoplasia type 4
MRI: magnetic resonance imaging
NGS: next-generation sequencing
PET: positron emission tomography
PHPT: primary hyperparathyroidism
rr: reference range
UFC: urinary free cortisol
